# An eCIRP inhibitor attenuates fibrosis and ferroptosis in ischemia and reperfusion induced chronic kidney disease

**DOI:** 10.1186/s10020-025-01071-2

**Published:** 2025-01-10

**Authors:** Fangming Zhang, Zhijian Hu, Asha Jacob, Max Brenner, Ping Wang

**Affiliations:** 1 Center for Immunology and Inflammation, Feinstein Institutes for Medical Research, Manhasset, NY USA; 2 TheraSource LLC, 350 Community Drive, Manhasset, NY USA; 3 Departments of Surgery and Molecular Medicine, Zucker School of Medicine, Manhasset, NY USA

**Keywords:** eCIRP, C23, Ischemia/reperfusion injury, Chronic kidney disease or CKD, Renal fibrosis

## Abstract

**Background:**

Chronic kidney disease (CKD) is a leading cause of death in the United States, and renal fibrosis represents a pathologic hallmark of CKD. Extracellular cold-inducible RNA-binding protein (eCIRP) is a stress response protein involved in acute inflammation, tissue injury and regulated cell death. However, the role of eCIRP in chronic inflammation and tissue injury has not been elucidated. We hypothesize that eCIRP is involved in renal ischemia/reperfusion (RIR)-induced CKD and that C23, an antagonist to eCIRP, is beneficial in attenuating renal fibrosis and ferroptosis in RIR-induced CKD.

**Methods:**

C57BL/6 (WT) or CIRP^−/−^ mice underwent renal injury with total blockage of blood perfusion by clamping bilateral renal pedicles for 28 min. In the WT mice at the time of reperfusion, they were treated with C23 (8 mg/kg) or vehicle. Blood and kidneys were harvested for further analysis at 21 days thereafter. In a separate cohort, mice underwent bilateral RIR and treatment with C23 or vehicle and were then subjected to left nephrectomy 72 h thereafter. Mice were then monitored for additional 19 days, and glomerular filtration rate (GFR) was assessed using a noninvasive transcutaneous method.

**Results:**

In the RIR-induced CKD, CIRP^−/−^ mice showed decreased collagen deposition, fibronectin staining, and renal injury as compared to the WT mice. Administration of C23 ameliorated renal fibrosis by decreasing the expression of active TGF-β1, α-SMA, collagen deposition, fibronectin and macrophage infiltration to the kidneys. Furthermore, intervention with C23 significantly decreased renal ferroptosis by reducing iron accumulation, increasing the expression of glutathione peroxidase 4 (GPX4) and lipid peroxidation in the kidneys of RIR-induced CKD mice. Treatment with C23 also attenuated BUN and creatinine. Finally, GFR was significantly decreased in RIR mice with left nephrectomy and C23 treatment partially prevented their decrease.

**Conclusion:**

Our data show that eCIRP plays an important role in RIR-induced CKD. Treatment with C23 decreased renal inflammation, alleviated chronic renal injury and fibrosis, and inhibited ferroptosis in the RIR-induced CKD mice.

## Background

Chronic kidney disease (CKD) affects 20 million people in the US. Managing CKD poses a significant socioeconomic burden worldwide, particularly for end-stage CKD patients requiring kidney replacement therapy through dialysis or transplantation. Tubulointerstitial fibrosis is a chronic and progressive condition affecting kidneys during CKD. Renal fibrosis and CKD affect up to 50% of adults above 70 years old and 10% of people worldwide (Glassock et al. 2017; Norregaard et al. 2023). There are currently FDA approved drugs against CKD associated with type 2 diabetes such as finerenone or SGLT2 inhibitors (Bakris et al. 2020; Neal et al. 2017; Perkovic et al. 2019; Wanner et al. 2016; Wiviott et al. 2019). Other drugs available are related to halting the progression of established CKD (Heerspink et al. 2020; Packer et al. 2020). However, there are no approved treatment for ischemia reperfusion (IR)-induced CKD. To date, clinical treatment strategies for IR-induced CKD have primarily focused on blood pressure control through the blockade of the renin-angiotensin system and glycemic control (Breyer and Susztak 2016). End-stage renal disease (ESRD) is typically addressed through dialysis and kidney transplantation, greatly increasing health and economic burden.

The pathological state of renal fibrosis is characterized by renal injury, inflammation, activation of myofibroblasts, and deposition of extracellular matrix (ECM) (Duffield 2014). The mechanism of renal fibrosis involves a variety of factors, including injury to renal tubular epithelial cells, infiltration of inflammatory cells and activation of myofibroblasts. Myofibroblasts are major cellular contributors to the deposition of ECM including collagen and fibronectin (Tomasek et al. 2002). TGF-β, Wnt, Notch and Hedgehog signaling pathways are considered as the major regulatory pathways in renal fibrosis (Wu et al. 2024; Grande et al. 2015; Lovisa et al. 2015). In addition, recent research has unveiled a connection among renal fibrosis, iron metabolic disorder, and ferroptosis. Ferroptosis, firstly proposed in 2012 (Dixon et al. 2012), is an iron-dependent form of regulatory cell death characterized by intracellular iron accumulation, lipid peroxidation and the loss of activity of the lipid repair enzyme GPX4 due to iron imbalance in cell metabolism (Liu and Wang 2022). Compelling evidence have indicated the role of ferroptosis in the pathophysiological process of injury in multiple tissues and organs. Inhibition of ferroptosis could decrease tissue injury and targeting ferroptosis provides new promising avenue for preventing the progression of kidney injury (Guo et al. 2023).

We have discovered that extracellular cold-inducible RNA-binding protein (eCIRP), a stress-response protein, acts as a new DAMP to trigger pro-inflammatory responses by binding the Toll-like receptor 4 (TLR4)/myeloid differentiation protein 2 (MD2) complex (Qiang et al. 2013), triggering receptor expressed on myeloid cells-1(TREM-1) (Denning et al. 2020) or IL-6R (Zhou et al. 2020). The role of eCIRP in acute inflammation, acute organ injury, and regulatory cell death (RCD) including apoptosis, pyroptosis, ferroptosis, and NETosis, has been studied (Aziz et al. 2019; Shimizu et al. 2022). We have discovered and developed eCIRP inhibitors to reduce acute inflammation, alleviate organ injury, and improve survival after acute injuries (Aziz et al. 2019; Zhang et al. 2018; Bolourani et al. 2021; Denning et al. 2019; McGinn et al. 2018). We have previously shown that deficiency in CIRP and blocking eCIRP with a neutralizing antibody attenuated inflammation and oxidative stress and lessened renal injury suggesting eCIRP as a target in the treatment of RIR injury (Cen et al. 2016). We have also previously shown in a mouse model of RIR followed by reperfusion for 24 h that C23, an eCIRP antagonist, significantly decreased renal inflammation and subsequent injury and showed a survival advantage up to 7 days (McGinn et al. 2018). However, little is known about eCIRP’s role in chronic injury including CKD. As such, we hypothesize that eCIRP is involved in renal fibrotic formation in RIR-induced CKD. We further propose that C23, can ameliorate RIR-induced renal fibrosis by reducing chronic renal inflammation, lessening renal fibrotic process, as well as reducing ferroptosis. To delineate the role of eCIRP in RIR-induced CKD, we implemented a 21-day chronic renal injury model using bilateral RIR in mice. First, we have shown that in this RIR-induced CKD model, renal injury and fibrosis was attenuated in the CIRP^−/−^ mice as compared to the WT mice. Next, treatment with C23 significantly decreased renal injury, renal fibrosis and ferroptosis in the WT RIR-induced CKD mice. Finally, glomerular filtration rate (GFR) was attenuated in the WT mice subjected to left nephrectomy 72 h after the bilateral RIR and C23 treatment prevented this decrease in GFR.

## Materials and methods

### Experimental animals

Male C57BL/6 (WT) mice, 6–8 weeks old (20–25 g) purchased from Charles River Laboratories (Wilmington, MA), and C57BL/6 CIRP^−/−^ mice originally obtained from Prof. Jun Fujita (Kyoto University, Kyoto, Japan), were housed in a temperature-controlled facility with a 12-hour light/dark cycle. The WT mice purchased from the vendor was acclimated to the environment for a minimum of 5 days prior to experiment as per the guide for the care and use of animals in research. The CIRP^−/−^ mice was bred in-house and they do not exhibit any distinct characteristics from the WT mice at the age that has been utilized for the study (i.e., 8–12 weeks old). All mice were fed a standard murine chow diet and had access to water *ad libitum*. The animal experiments were conducted in accordance with the recommendations of the National Institutes of Health guidelines for the use of experimental animals and was approved by the Institutional Animal Care and Use Committee (IACUC) of the Feinstein Institutes for Medical Research. The study was conducted in compliance with the ARRIVE guidelines (checklist provided).

### Peptide synthesis

*C23* (GRGFSRGGGDRGYGG) was synthesized by GenScript USA Inc. (Piscataway, NJ), purified to > 95% by HPLC, and provided as a lyophilized powder. The peptide was then resuspended to desired concentration in normal saline prior to use in mice (Qiang et al. 2013).

### Bilateral renal ischemia reperfusion (RIR) injury

A total of 9 WT and 9 CIRP^−/−^ mice were included in the study. The WT and CIRP^−/−^ mice were randomly divided into 4 mice as shams and 5 mice as RIR for each strain. The mice were anesthetized with 1–2% isoflurane inhalation. A midline incision of approximately 1.5 cm was made after shaving the abdomen and prepping it with 10% povidone-iodine and 75% alcohol. For the bilateral RIR model, microvascular clamps were used to occlude the vascular pedicles of both kidneys. The clamps were removed after 28 min to allow reperfusion. The sham mice underwent the same procedure, with the exception that the vascular pedicles were not clamped. Mice in all groups were then subcutaneously injected with 1 ml of normal saline for resuscitation after the closure of the abdomen. After surgery and recovery, the mice were then returned to their home cages and received standard murine chow diet and water *ad libitum*. Mice were monitored once daily for the first three days post-operatively and then monitored three times per week. In addition, the mice were weighed at least once within 48 h and then twice weekly. The monitoring of mice involved strict accordance for the humane endpoints. The humane endpoint is defined as the point at which death is imminent or suffering is irreversible. The criteria utilized were the following: [1] Activity level is minimal or absent; [2] Weight loss of ≥ 20%; [3] Hunched or recumbent posture; [4] Minimal or no response to stimuli; [5] Grimace = 2; [6] Body conditioned score ≤ 2. The presence of two or more of these criteria were considered as fulfilling the criteria for early euthanasia. At 21 days after the bilateral RIR, mice were euthanized using CO_2_ asphyxiation followed by exsanguination. Kidneys were collected from all groups of mice. The left kidney from each mouse was preserved in 10% formalin for histopathological analysis, while the right kidney was flash-frozen in liquid nitrogen and stored at -80 °C for protein analysis.

### RIR injury and administration of C23

A total of 22 WT mice were included in the study. The mice were randomly divided into 4 mice as shams, 8 mice as RIR treated with C23 and 10 mice as RIR treated with vehicle (normal saline). The bilateral RIR was conducted as above and C23 at a dose of 8 mg/kg, resuspended in 100 µl normal saline, was intraperitoneally administered immediately after the clamp removal to the mice in the RIR + C23 group. In the sham and RIR mice, 100 µl of normal saline was administered instead. After surgery and recovery, the mice were returned to their home cages and monitored as above. At 21 days after the bilateral RIR, mice were euthanized using CO_2_ asphyxiation followed by exsanguination. Blood and kidneys were harvested. Blood samples were centrifuged at 1,000 x g for 10 min at 4 °C, and the resulting serum samples were stored at -80 °C until assayed. The kidneys were separated, processed, and stored as above for further analysis.

### Renal function measurement

Glomerular filtration rate (GFR) was conducted as a measure of in vivo renal function. We first estimated GFR in the 21-day post-RIR mice but the results showed that while BUN and creatinine were significantly increased, there was no substantial changes in GFR in those mice compared to sham operated mice. We suspected that the lack of changes in GFR could be due to the less severity of the bilateral RIR model. Therefore, a separate cohort of mice were subjected to bilateral RIR and 72 h later, they underwent left nephrectomy. A total of 27 WT mice were included in the study. The mice were randomly divided into 7 mice as shams, 10 mice each as RIR treated with C23 and RIR treated with vehicle (normal saline). RIR mouse was either treated with C23 or vehicle at the time of reperfusion, which would have been two days prior to the nephrectomy. Sham mice underwent similar procedure apart from RIR and nephrectomy. GFR was measured in conscious mice 21 days after the bilateral RIR (Hu et al. 2023). Briefly, a small fluorometer (MediBeacon, St. Louis, MO, USA) was affixed to the shaved flank of the mice. Subsequently, 0.15 mg/kg of FITC-sinistrin (Thermo Fisher Scientific, Fair Lawn, NJ, USA) in 100 µl of normal saline was intravenously injected via the retro-orbital sinus. The fluorometer was programmed to make transcutaneous measurements every 2 s, for up to 90 min. The device was removed from the mice and the mice were euthanized and discarded. No other measurements were done in this cohort. Data that were automatically stored on the device were analyzed using the imaging device MPD Studio software (MediBeacon). GFR in µl/min/100 g body weight was calculated from the decrease in fluorescence intensity of FITC-sinistrin, following the manufacturer’s instructions in the software.

### Determination of serum markers of renal function

Serum levels of blood urea nitrogen (BUN; Ref # B7552-450) and creatinine (Cat # C7530-150) were determined using colorimetric assay kits (Pointe Scientific, Canton, MI, USA) following the manufacturer’s protocol.

### Histology and immunostaining

The formalin-fixed kidney was embedded in paraffin, sectioned into 5-µm slices, dehydrated, and then stained with H&E, Masson’s trichrome, IHC, and Perl’s. For H&E staining, the tissue section was examined using a light microscope at 200× magnification. Injury was assessed in a blind fashion by semi-quantitation based on our previous publications ((Cen et al. 2016; Hu et al. 2023). Scores were averaged for each sample over 5 randomly selected fields. For Masson’s Trichrome staining (collagen, blue color) in the kidney, the tissue slice was examined using a light microscope at 100x magnification. Measurements were conducted using ImageJ software, and the blue color density was calculated as the mean from five individual fields for each slice. For immunohistochemistry staining, the sections were blocked with 2% H_2_O_2_ in 60% methanol at room temperature for 10 min. Subsequently, they were incubated overnight at 4 °C with the diluted primary antibodies against fibronectin (1:200) (Cell Signaling Technology, Danvers, MA, USA), α-SMA (1:200) (Cell Signaling) and F4/80 (1:200) (Cell Signaling). After incubation with appropriate secondary antibodies, the sections were developed with DAB and then counterstained with hematoxylin. The section was examined using a light microscope at 200x magnification, and the quantification was performed using Image J software. Perls’ Prussian blue stain was performed using an iron stain kit (Polysciences, Inc., Warrington, PA). Deparaffinized kidney sections were incubated in freshly prepared Perls’ solution (1:1 mixture of 4% potassium ferrocyanide and 4% hydrochloric acid) at 37 °C for 1 h, followed by rinsing with distilled water. They were then counterstained with nuclear fast red for 3 min, rinsed, dehydrated, and covered. The sections were observed under a light microscope at 400x magnification. The blue-stained areas were counted in a blind fashion.

### Western blotting

The frozen kidney tissues were powdered in liquid nitrogen and homogenized in RIPA lysis buffer and protein concentrations estimated using the Bio Rad protein assay. Then, the protein lysates (50 µg) were separated by SDS-PAGE and transferred to nitrocellulose membranes. After overnight incubation at 4 °C with primary antibodies, including TGF-β1 (1:1,000) (Cell Signaling), GPX4 (1:500) (Cell Signaling), and β-actin (1:10,000) (Sigma-Aldrich, Louis, MO, USA), the membranes were then incubated with an HRP-conjugated secondary antibody (1:10,000) (Amersham, Little Chalfont, UK) for 1 h. The bands were detected with ECL. The membranes were scanned in the Odyssey image system (LI-COR) and densitometric analysis was quantitated using ImageJ software.

### MDA assay

The MDA concentration of kidney tissue lysates was assessed using the malondialdehyde (MDA) assay kit (Cat #MAK085; Sigma-Aldrich) according to the manufacturer’s instructions. The lysates were mixed with thiobarbituric acid, heated at 95^o^C for 60 min, and cooled to room temperature for 10 min. The lysates were then measured at absorbance 532 nm using the BioTek Synergy Neo2 microplate reader (Agilent). The MDA concentration was calculated as nmol/mg tissue.

### Statistical analysis

The data are expressed as mean ± SEM and analyzed using one-way ANOVA, followed by Student Newman Keul’s (SNK) post-hoc test analysis for comparisons among multiple groups. Given the limited number of mice in the study, it is more appropriate not to assume normality. Therefore, duplicate analysis was performed using non-parametric statistical tests, i.e., Kruskal-Wallis test. The statistical outcomes were same as what were already observed with one-way ANOVA and SNK test. *P* < 0.05 were considered statistically significant. All data analyses were conducted using GraphPad statistical program and software (GraphPad, Boston, MA).

## Results

### eCIRP is implicated in renal fibrosis in RIR-induced CKD mice

**Fig. 1:**
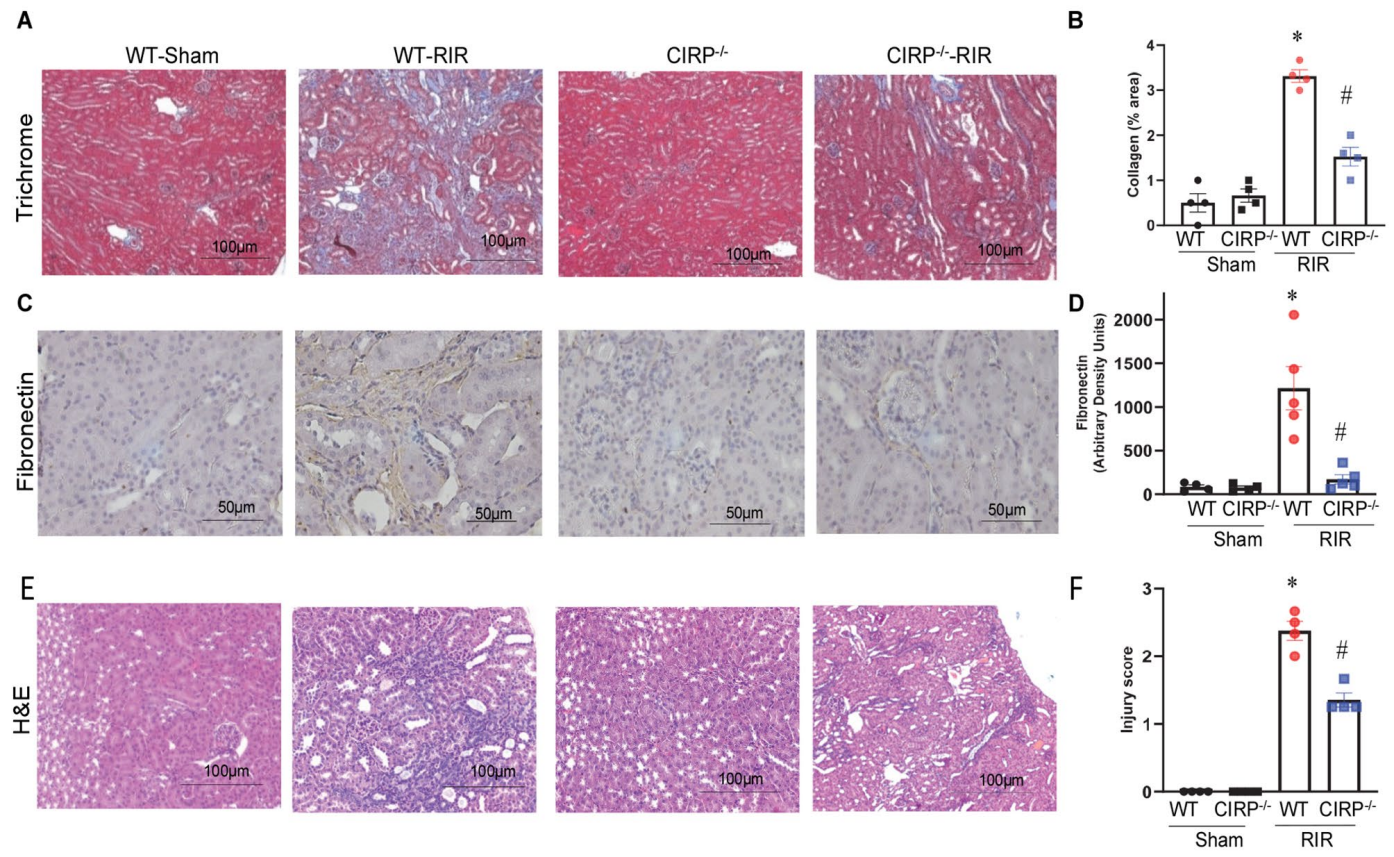
*CIRP is involved in renal fibrosis in RIR-induced CKD mice*. Wild-type (WT) and CIRP^−/−^ male C57BL/6 mice were subjected to renal ischemia and reperfusion (RIR). On 21 days after RIR, kidneys were harvested from both sham and RIR mice. Renal tissue was stained using Masson’s Trichrome method (**A**), and the area in percentages of collagen stained as blue in color was determined using ImageJ (**B**). Renal tissue sections were analyzed by immunohistochemistry for fibronectin (**C**) and quantified by scoring the intensity of the brown color using ImageJ (**D**). Renal tissue sections were stained with Hematoxylin & Eosin and representative images at 200x magnification are shown (**E**) and the injury score was quantified (**F**). The data are expressed as mean ± SE (*n* = 4–5/group) and analyzed by one-way ANOVA and the SNK method and using Kruskal-Wallis (non-parametric) test. *P < 0.05 vs. Shams; ^#^*P* < 0.05 vs. WT RIR

To determine whether CIRP is involved in renal fibrosis, we subjected WT and CIRP^−/−^ mice to RIR and kidney sections of 21-day post-RIR mice were stained using Masson’s Trichrome staining. The results showed that the blue stain indicative of collagen deposition was 6.0-fold higher in the WT mice than those in the shams. However, the staining was 35% lower in the CIRP^−/−^ mice than in the WT mice (Fig. 1A-B). Fibronectin, an indicator to the presence of myofibroblast, was also evaluated using IHC staining. The results indicated that fibronectin staining was increased in the WT mice 21 days after RIR. In contrast, fibronectin staining was significantly decreased in the CIRP^−/−^ mice than in the WT mice (Fig. 1C-D). These results suggest that eCIRP plays an important role in RIR-induced renal fibrosis. Histological staining with H&E in the 21-day post-RIR mice of WT and CIRP^−/−^ indicate that while there was increased injury in the WT RIR mice, the injury was significantly decreased in the CIRP^−/−^ RIR mice (**Fig. IE-F**).

### C23 ameliorated renal injury

**Fig. 2:**
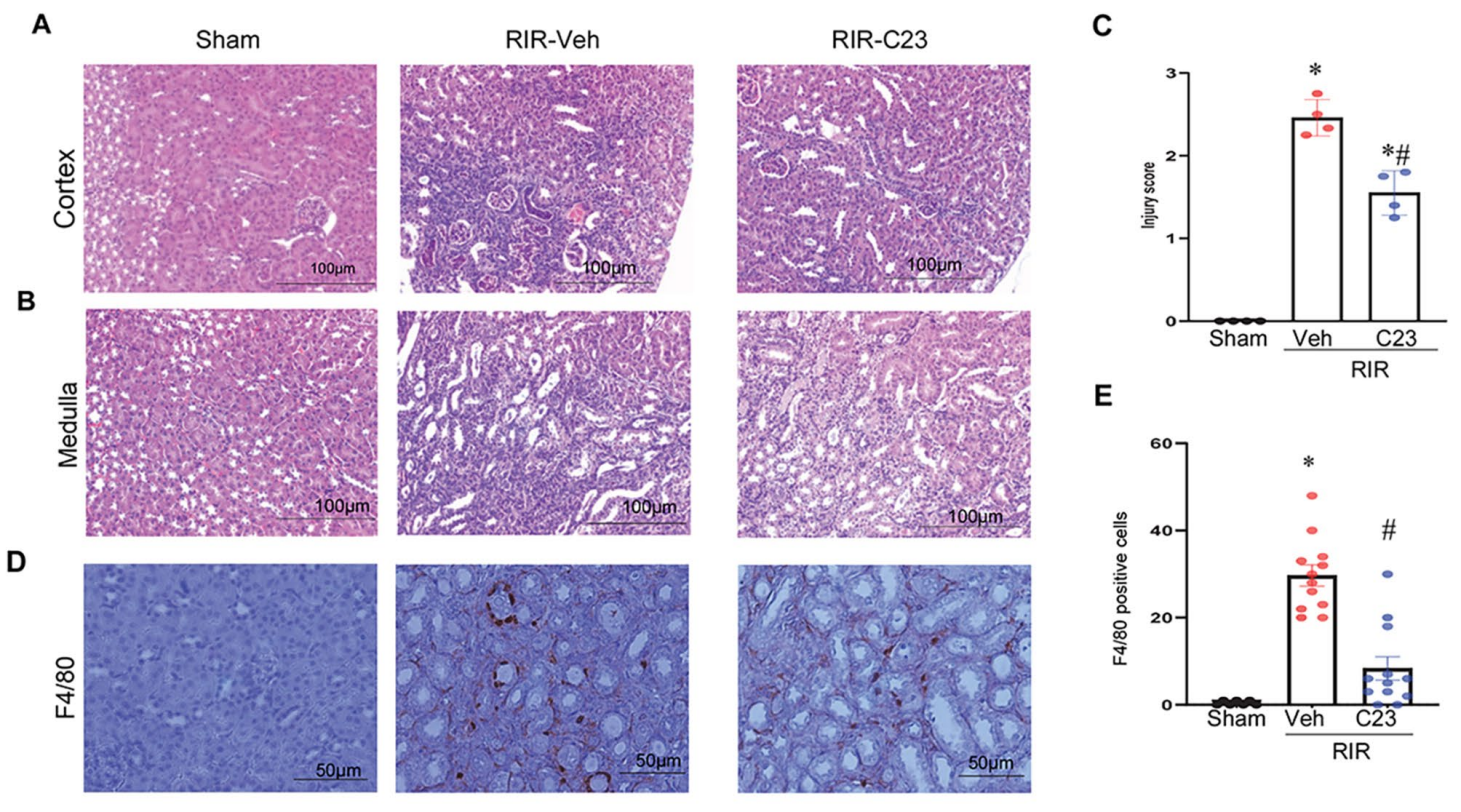
*C23 reduced renal injury and macrophage infiltration in RIR-induced CKD mice*. Renal tissues harvested from sham mice and the 21days post-RIR mice treated with C23 or vehicle at the time of reperfusion were sectioned and stained with hematoxylin-eosin (H&E). Representative photomicrographs of the cortex (**A**) and medulla (**B**) at 200x magnification are presented and the histologic injury scores (**C**) are shown. Renal tissues were sectioned and stained with F4/80 antibody (**D**) and quantified using ImageJ (**E**). Data are expressed as means ± SEM (*n* = 4–7/group) and compared using one-way ANOVA and the SNK method and using Kruskal-Wallis (non-parametric) test; *P < 0.05 vs. Sham; ^#^*P* < 0.05 vs. RIR-Veh

Inflammation can cause tissue damage and promote the development of tissue fibrosis. Simultaneously, scar tissues can trigger an inflammatory response. To assess the impact of C23 administration on renal injury induced by RIR-induced CKD, we evaluated renal histologic damage using H&E staining. Figure 2A (cortex) and **2B** (medulla) showed that the renal tissues in the 21-day post-RIR-vehicle mice exhibited severe tubular cellular necrosis, tubular dilation, cast formation, and inflammatory cellular infiltration. A histologic damage score increased in the RIR-vehicle mice compared to the shams, while it decreased significantly by 37% in the C23 treated RIR mice (Fig. 2C). A parallel dramatic increase in macrophage cell infiltration was observed in the RIR-vehicle mice as evidenced by the F4/80 staining. Importantly, C23 significantly reduced this infiltration by 72% (Fig. 2D-E). Therefore, RIR caused significant histologic damage and macrophage infiltration in the kidney tissue, which was prevented by C23 treatment.

### C23 suppressed renal fibrosis

**Fig. 3:**
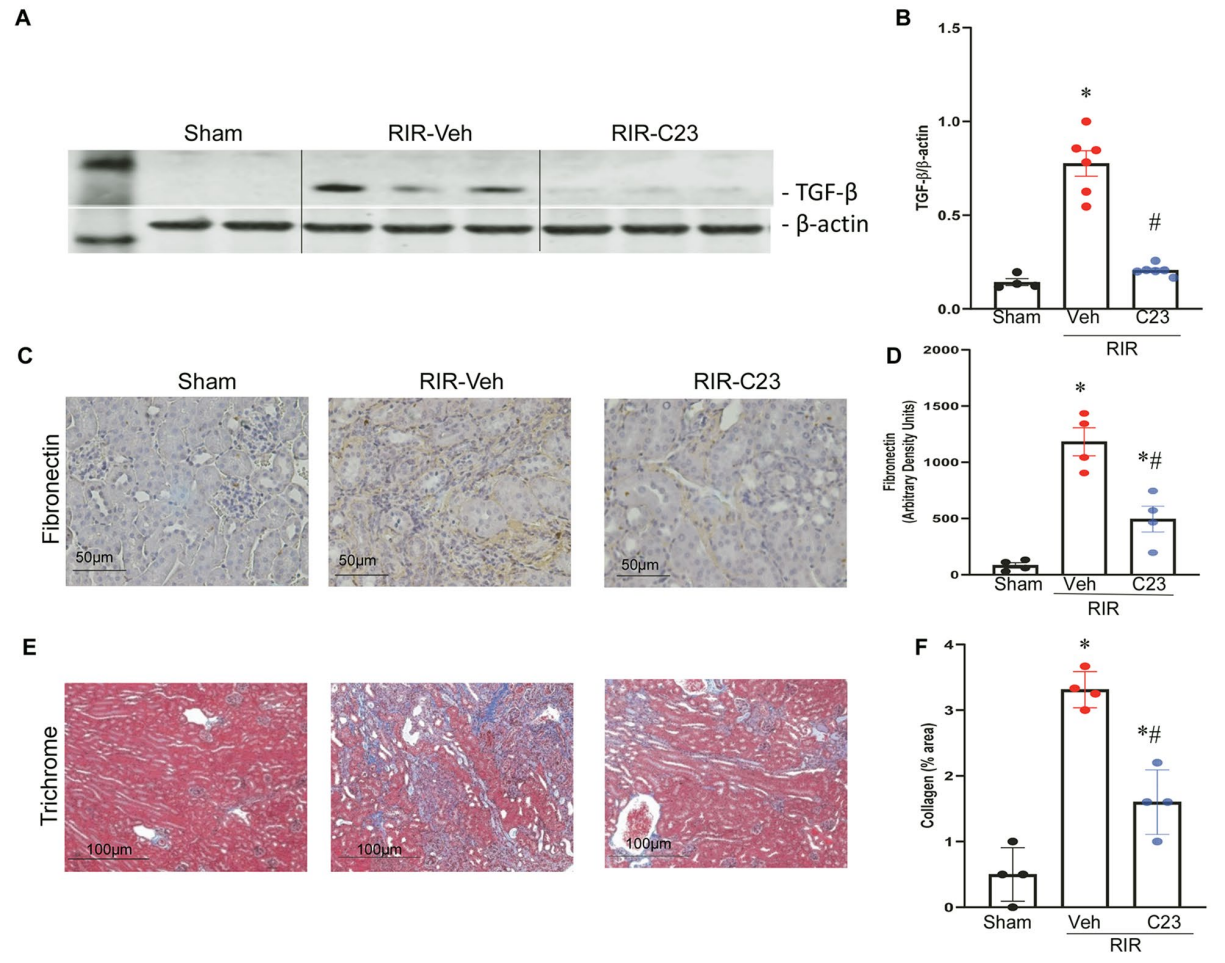
*C23 improved renal fibrosis in RIR-induced CKD mice*. Renal tissues from different groups were subjected to protein extraction and lysates were analyzed for TGF-β1 protein expression using Western blotting. The representative blots were scanned, shown as cropped gel images (**A**) and quantified by densitometry (**B**). The band intensity of active TGF-β1 was normalized to the corresponding band intensity of β-actin. Renal tissue sections were analyzed by immunohistochemistry for fibronectin (**C**) and quantified by scoring the intensity of the brown color using ImageJ (**D**). Renal tissue sections were stained using Masson’s Trichrome (**E**), and the percent areas of collagen stained in blue color was determined with ImageJ (**F**). Data are expressed as means ± SEM (*n* = 4–6/group) and compared by one-way ANOVA and the SNK method and using Kruskal-Wallis (non-parametric) test; *P < 0.05 vs. Sham; #*P* < 0.05 vs. RIR-Veh

Transforming growth factor-β1 (TGF-β1), produced by renal inflammatory cells during acute and chronic inflammation plays a crucial role in promoting fibrosis by inducing the differentiation of quiescent cells into matrix-secreting myofibroblasts (Meng et al. 2016). To assess the impact of C23 intervention on renal fibrotic formation, we initially examined the expression of TGF-β1 in the kidneys of 21-day post-RIR mice. The Western blot data revealed that the expression of activated TGF-β1 (25 Kd) was significantly upregulated in the RIR-vehicle mice compared to the shams. However, administration of C23 markedly attenuated the expression of activated TGF-β1 by 73% (Fig. 3A-B). We also evaluated fibronectin expression in renal tissue using IHC staining. The results affirmed that the expression of fibronectin significantly upregulated in the RIR-vehicle mice. However, the expression of fibronectin is significantly lowered by 58% in the C23 treated RIR mice compared to that in the RIR-vehicle mice (Fig. 3C-D). We simultaneously examined the kidney sections after staining with Masson’s trichrome. The blue stain, indicative of collagen deposition, was significantly increased in the RIR-vehicle mice compared to the shams. In contrast, treatment with C23 significantly reduced the trichrome staining by 52% (Fig. 3E-F). These results demonstrated that C23 treatment reduced renal profibrotic factors and collagen deposition, thereby improving renal fibrosis in this chronic renal injury model of RIR-induced CKD.

### C23 attenuated myofibroblast differentiation and renal ferroptosis

**Fig. 4:**
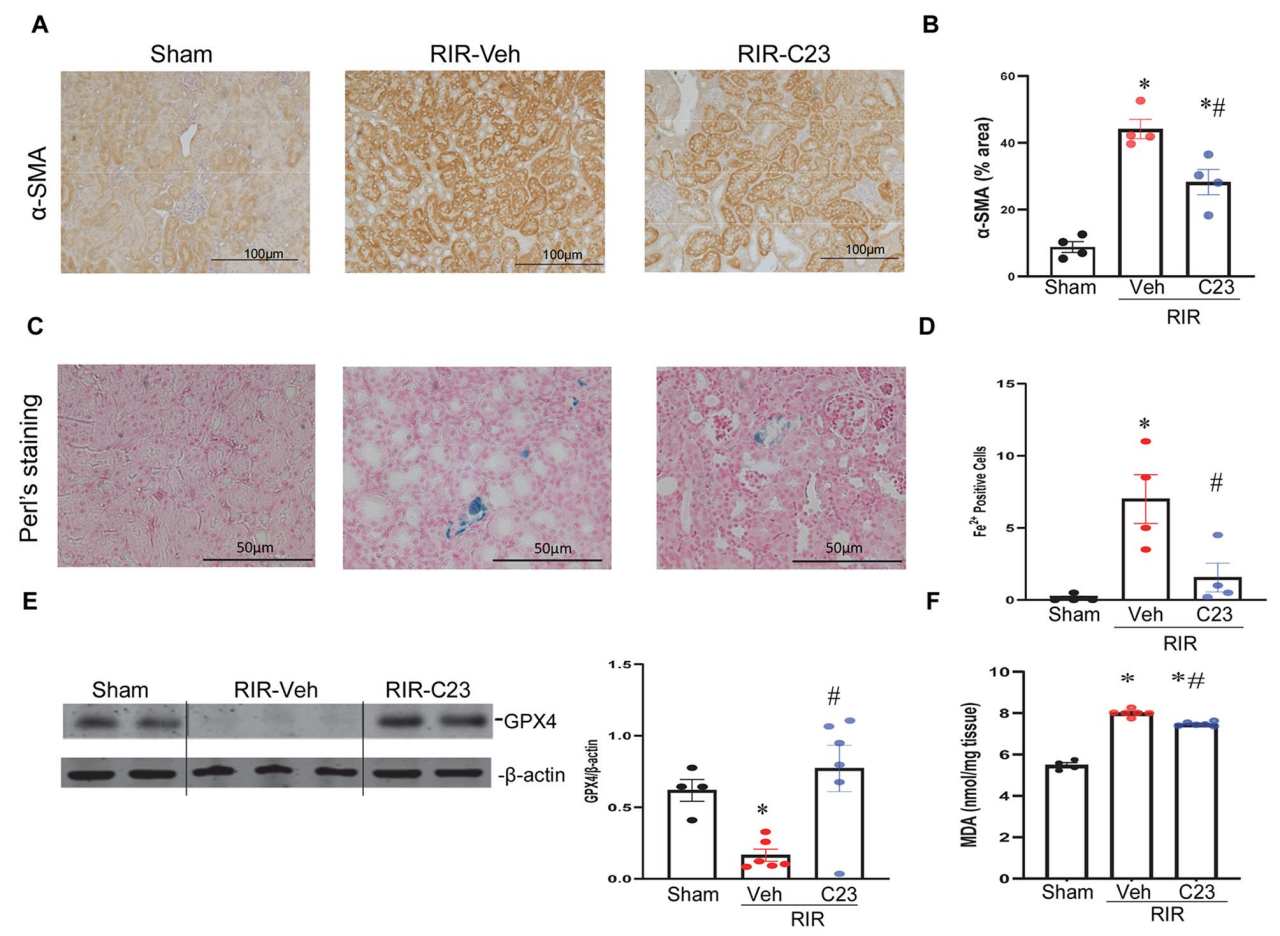
*C23 reduced myofibroblast formation and ferroptosis in the kidneys in RIR-induced CKD mice*. Renal tissues were sectioned and analyzed by immunohistochemistry for α-SMA (**A**) and quantified using ImageJ (**B**). Renal tissue sections were stained using Perl’s for iron deposition assessment. Representative photomicrographs at 400x magnification are shown (**C**). The blue intensity was determined using the imageJ software (**D**). Protein lysates from the different groups underwent Western Blotting. The blots were scanned, shown as cropped gel images and quantified by densitometry (**E**). The band intensity of GPX4 was normalized to the corresponding band intensity of β-actin. Protein lysates from the different groups were measured for the levels of lipid peroxidation using a MDA kit (**F**). Data are expressed as means ± SEM (*n* = 4–6/group) and compared by one-way ANOVA and the SNK method and using Kruskal-Wallis (non-parametric) test; *P < 0.05 vs. Sham or control; ^#^*P* < 0.05 vs. RIR-Veh

Similar to smooth muscle cells, myofibroblasts are capable of contraction due to the presence of cytoskeletal proteins, mainly α-SMA (Klingberg et al. 2013). α-SMA is upregulated in wound scars and fibrotic tissues. To assess the potential of C23 to alleviate myofibroblast activation, we examined the expression of α-SMA using IHC staining in the kidneys. The results showed that α-SMA expression in renal tissues significantly increased by 5-fold in the RIR-vehicle mice compared with that in the shams. In contrast, intervention with C23 markedly reduced expression by 44% in the C23 treated RIR mice compared to the RIR-vehicle mice (Fig. 4A-B). Ferroptosis is involved in the progression of fibrotic diseases and plays a key role in renal fibrosis. Overload of iron levels in tissues is a characteristic of ferroptosis. To determine whether C23 ameliorated ferroptosis in RIR-induced injury, we initially investigated iron-positive cells in renal tissues using Perl’s Prussian blue staining. The results revealed a significant increase in iron overload in the renal tissue of the RIR-vehicle mice compared to the shams. However, C23 treatment led to a significant 78% reduction in the number of renal iron-positive cells compared to the RIR-vehicle mice (Fig. 4C-D). GPX4 is the central regulator of ferroptosis, and a decrease in GPX4 activity is often used as a marker of ferroptosis. In the current study, we assessed GPX4 expression in the kidneys using Western blotting. The results revealed a significant 74% decrease in GPX4 expression in the RIR-vehicle mice compared to the shams. Treatment with C23 significantly attenuated the downregulation of GPX4 expression from RIR-vehicle mice and restored them to sham levels (Fig. 4E). Since a hallmark of ferroptosis is lipid peroxidation, we have determined the levels of malondialdehyde (MDA), an indicator of lipid peroxidation. The results showed that MDA was increased by 1.4-fold in the kidney tissues of RIR-vehicle mice as compared to sham mice and C23 treatment significantly decreased these levels (Fig. 4F). In summary, C23 had a beneficial effect in ameliorating ferroptosis.

### C23 improved serum markers of renal function in 21 days post-RIR mice

**Fig. 5:**
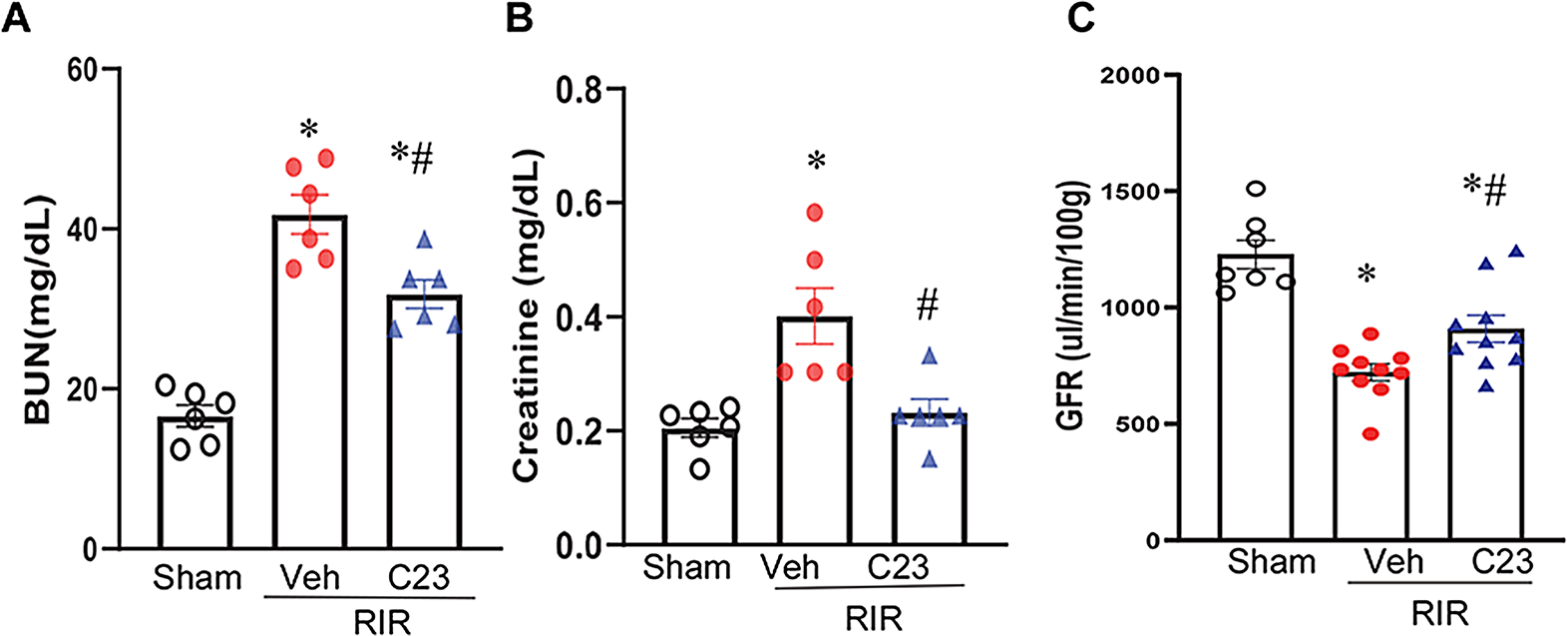
C23 attenuated serum markers of renal function in RIR-induced CKD mice and attenuated GFR in mice which underwent left nephrectomy after RIR. Blood was collected from different groups in mice from the experiments in Fig. 2 and serum levels of blood urea nitrogen (BUN) (**A**) and creatinine (**B**) were measured. The data are expressed as mean ± SEM (*n* = 6/group). Male C57BL/6 mice were subjected to renal ischemia and treated with C23 (8 mg/kg) or vehicle at the beginning of the reperfusion. At 72 h after RIR, mice underwent left nephrectomy and then monitored. On 21 days after RIR, glomerular filtration rates (GFRs) were assessed using a noninvasive transcutaneous device (**C**). The data are expressed as means ± SEM (*n* = 7–10/group) and compared using one-way ANOVA and the SNK method and using Kruskal-Wallis (non-parametric) test; *P < 0.05 vs. Sham; ^#^*P* < 0.05 vs. RIR-Veh

To further delineate the role of eCIRP in renal fibrosis, WT mice subjected to RIR and treated with C23 or vehicle were examined for serum markers of renal function in 21-days post-RIR mice. Blood urea nitrogen (BUN) and creatinine are conventional serum biomarkers for renal function. To determine the effect of C23 on renal function, we examined serum levels of BUN and creatinine. The serum levels of BUN and creatinine in the vehicle mice remained higher compared to those of the shams, and treatment with C23 significantly decreased the serum levels of BUN and creatinine by 24% and 42%, respectively (Fig. 5A-B). The data suggest that C23 has beneficial effects in renal function in RIR-induced CKD mice.

### C23 improved GFR in mice which underwent both RIR and left nephrectomy

GFR is an important in vivo clinical indicator of kidney function. In a separate cohort, mice were subjected to bilateral RIR and treated with C23 or vehicle. At 72 h after RIR, they underwent left nephrectomy. The mice were then monitored for additional 19 days, and GFR was measured. GFR was considerably decreased by 41% in the RIR-vehicle mice as compared to the shams whereas administration with C23 significantly increased the GFR by 26% from RIR-vehicle mice (Fig. 5C).

## Discussion

In the current study, we first found the involvement of CIRP in the fibrotic process by showing that the deficiency in CIRP protects animals from RIR-induced renal fibrotic injuries. Treatment with C23 at the time of reperfusion significantly decreased renal injury which led to a reduction in fibrosis 21 days after RIR. The mechanism of renal fibrosis involves a variety of factors, including injury to renal tubular epithelial cells, infiltration of inflammatory cells and activation of myofibroblasts. TGF-β signaling pathway is considered as the major regulatory pathways in renal fibrosis (Wu et al. 2024). Our results clearly showed that the amount of active TGF-β1 protein expression was significantly higher in the kidneys of the 21-day post RIR mice compared to that in the sham group. It is noteworthy that administration with C23 dramatically downregulated the expression of active TGF-β1 protein. However, studies have shown that targeted disruption of TGF-β1 gene in mice resulted in the development of massive multifocal inflammatory disease suggesting that immune dysregulation is a possible mechanism for the inflammatory diseases (Yaswen et al. 1996). Since TGF-β1 has shown to be anti-inflammatory, one can argue that its inhibition by C23 could rather provoke inflammation than inhibit it. However, a large body of literature support that TGF-β1 is a major regulator of the induction of epithelial mesenchymal transition (EMT) and renal fibrosis (Lovisa et al. 2015; Xu et al. 2009). EMT is a cellular process in which cells acquire a mesenchymal phenotype and behavior after epithelial cell function is reduced. Although these observations indicate TGF-β1 could be directly targeted therapeutically for renal fibrosis, TGF-β1 regulates multiple biological responses in addition to fibrosis including cell proliferation, apoptosis, differentiation, autophagy, and the immune response (Bottinger and Bitzer 2002; Meng et al. 2013). Therefore, while targeting TGF-β1 directly is not a viable therapy, decrease in TGF-β signaling has been shown to be protective in renal diseases. Resveratrol, a polyphenol derived from grapes, can prevent EMT by modulating the signaling of the TGF-β/Smad axis and alleviating renal injury and fibrosis (de Jesus Soares et al. 2007). Therefore, it is plausible that inhibiting the TGF-β by C23 can elicit protection in RIR-induced renal fibrosis by preventing EMT induction. However, additional experiments are needed to directly show C23 possess anti-inflammatory effects while inhibiting TGF-β expression.

In the current study, treatment with C23 significantly reduced α-SMA expression, a marker of myofibroblast, in the kidneys of the 21-day post RIR mice. It appears that α-SMA staining was predominately present in the tubules which differs from the normal distribution of α-SMA in fibrotic kidneys in which it is localized to the interstitial space. While the origin of myofibroblast in the kidneys remains unknown, renal tubular epithelial cells have been implicated as a major source (Sheng and Zhuang 2020; LeBleu et al. 2013; Lin et al. 2008; Carew et al. 2012; Qi and Yang 2018). Renal fibrosis is closely associated with tubular cell injury. EMT of the renal epithelial cells is a major contributor to the development of renal fibrosis. Cells undergoing EMT express α-SMA among other markers, vimentin, collagen I and fibronectin. The change in these markers is indicative of EMT, demonstrating that the cells have acquired a fibroblast-like or myofibroblast like phenotype, thereby enhancing proliferative and invasive capacities (Li et al. 2023). EMT activation in the renal tubular epithelium is an intermediate step and therefore, reversing EMT can prevent renal fibrosis. In fact, in a TGF-β-induced HK-2 cells (human renal tubular epithelial cell line) as well as in the kidneys of a unilateral ureter obstruction (UUO) model, α-SMA was increased which suggested that EMT of the renal tubular epithelial cells leads to renal fibrosis (Yu et al. 2025). Therefore, increased α-SMA staining in the tubules suggests that in RIR, there is a substantial increase in EMT of the renal tubular epithelium presumably due to increase in TGF-β expression. Treatment with C23 significantly attenuated these increases in EMT indicating that C23 protects against eCIRP-induced EMT in RIR associated renal fibrosis. Collagen and fibronectin, two major components of the ECM molecules in fibrotic tissues, are widely used to assess the degree of fibrosis. Myofibroblasts produce large amounts of ECM molecules such as collagen and fibronectin. In addition to α-SMA, collagen I as measured by the Masson’s trichrome staining, and fibronectin are also increased in the kidneys of RIR mice. Treatment with C23 greatly reduced the expression of collagen, and fibronectin in the 21-day post-RIR kidneys. Therefore, intervention with C23 ameliorated chronic RIR-induced renal fibrosis by reducing collagen, and fibronectin. Excessive ECM deposition can lead to decreased kidney function.

Certainly, renal inflammation is a major contributor to renal fibrosis. Previously we have examined serum conventional markers such as TNF-α, IL-1β and IL-6 in both RIR mice and C23 treated RIR mice at 24 h after reperfusion (McGinn et al. 2018). Serum levels of these cytokines were significantly decreased in C23 treated RIR mice as compared with vehicle treated RIR mice. Likewise, mRNA expressions of TNF-α, KC, KIM-1 and NGAL were significantly decreased in C23 treated RIR mice as compared to vehicle RIR mice (McGinn et al. 2018). However, it is highly unlikely that these conventional inflammation markers would remain high in the 21-day post-RIR mice. Therefore, in the current study, we have examined the number of F4/80 staining cells indicative of macrophage infiltration to the kidneys in 21 days post-RIR mice. The F4/80 staining was increased in the RIR-vehicle mice and the staining was significantly attenuated with C23 treatment. Although the GFR remained unchanged in the bilateral RIR only model, GFR was decreased in 21 days later in a more severe model of post-RIR and left nephrectomy indicating decreased renal function. Treatment with C23 significantly improved renal function as shown by increase in GFR from the RIR-vehicle mice. Thus, eCIRP plays a crucial role in chronic injury and renal dysfunction in this RIR-induced CKD model.

Recent research has unveiled a connection among renal fibrosis, iron metabolic disorder, and ferroptosis. Ferrostatin-1 (Fer-1), a selective inhibitor of ferroptosis, has emerged as a significant factor in its treatment. However, researchers have noted that due to plasma and metabolic instability, the function of Fer-1 is weak in vivo (Friedmann Angeli et al. 2014; Zilka et al. 2017). Consequently, there is a need for a more stable and potent ferroptosis inhibitor in the field. Shimizu and colleagues demonstrated that C23 treatment inhibits the downregulation of GPX4 expression and upregulation of ROS in the mice lungs during sepsis (Shimizu et al. 2022). In fact, rmCIRP significantly decreased GPX4 expression in macrophages and increased lipid ROS in a TLR4 dependent manner (Shimizu et al. 2022). While other markers exist for ferroptosis (Sui et al. 2021), the loss of activity of the lipid repair enzyme GPX4 due to iron imbalance in cell metabolism is a hallmark indicator of ferroptosis. In our study, intervention of C23 decreased ferroptosis in RIR-induced renal injury, as evidenced by a reduction in iron-positive cells, an increase in GPX4 and a decrease in lipid peroxidation. It appears that much of the Perl’s Prussian blue stain which is indicative of iron content is present in the lumen rather than in cells. However, in kidney tissue sections obtained from RIR mice at 48 h after reperfusion, the blue stain was most clearly seen in the cells and was more prominent than that observed in the 21-day post-RIR samples (unpublished observation). This difference in the staining pattern between the 48 h and the 21-day post-RIR samples could be due to leaks in the tubular epithelium caused by chronic injury and maladaptive repair process of the injured epithelium in the 21-day post-RIR samples. Nevertheless, ferroptosis is closely linked to renal fibrosis through shared metabolic pathways and pathological mechanisms (Guo et al. 2023), and has been implicated in the development of renal fibrosis in distinct experimental animal models, such as UUO (Zhang et al. 2021) and RIR (Andre et al. 2023; Ide et al. 2021). Meanwhile, ferroptosis inhibitors can prevent or delay renal fibrosis and mediate interstitial fibroblasts’ fibrotic response (Guo et al. 2023). Our results demonstrate the simultaneous presence of ferroptosis and fibrosis in the kidneys of the 21-day post RIR mice. Administration of C23 not only improved the renal fibrotic process but also suppressed ferroptosis, supporting the theory that ferroptosis plays a role in tissue fibrotic constitution. Additional experiments are needed to address additional mechanisms by which eCIRP causes renal fibrosis and how C23 blocks this effect in this RIR-induced CKD model.

The mechanism by which eCIRP facilitates RIR-induced renal fibrosis and that how C23 inhibits this process has not been completely elucidated. CIRP is an evolutionarily conserved RNA chaperone. It is predominately present in the nucleus of every cell, and it regulates mRNA transcription and processing. During cellular stress and inflammatory conditions, CIRP translocates from the nucleus to the cytoplasm and then released to the extracellular space. In this regard, we have previously examined the expression of CIRP in the kidneys and serum at 5 h and 24 h after bilateral RIR, same as the model described in the current study. In renal tissue, CIRP was not changed at 5 h but increased by 42% at 24 h compared to sham controls. In the serum, eCIRP was barely detected at 5 h but present at 24 h after RIR (Cen et al. 2016). Although macrophages are the primary source in upregulating CIRP due to cellular stress, CIRP is present in nearly all cells and upon cell death possibly by necrosis or pyroptosis, it can be released into the extracellular space. Therefore, it is difficult to ascertain which cell type in the kidney is responsible for its expression and subsequent release into circulation.

We have previously identified TLR4 as the primary receptor for eCIRP and thus characterized it as a DAMP (Qiang et al. 2013). Single administration of rmCIRP in vivo induced acute kidney injury as evidenced by increased serum levels of BUN, creatinine and serum and urine levels of NGAL (Siskind et al. 2022). Since eCIRP promotes inflammation by TLR4 signaling, eCIRP could initiate the inflammatory cascade as early as 24 h after RIR leading to recruitment of monocytes/macrophages and activate tissue resident dendritic cells, fibroblasts, and other tubular epithelial cells. These activated cells release proinflammatory cytokines, chemokines, and reactive oxygen species that further damage tubular epithelial cells and peritubular capillary endothelial cells, causing them to undergo ferroptosis, the primary regulated cell death in AKI, and shed into the tubular lumen (Tang et al. 2019). The ensuing obstructive (cellular and granular) tubular casts and capillary obliteration elicit tubular dysfunction. The surviving tubular epithelial cells then undergo dedifferentiation and proliferate to repair the damaged tubular epithelium. However, persistent inflammation and an incomplete or maladaptive repair process induce the transformation of fibroblasts, macrophages, and other cells into myofibroblasts (Grande et al. 2015; Ide et al. 2021; Sato and Yanagita 2018; Lever et al. 2019; Kuppe et al. 2021), which deposit large amounts of extracellular matrix proteins such as collagen and vimentin (Sato and Yanagita 2018; Lever et al. 2019; Falke et al. 2015). The resulting tubulointerstitial fibrosis, renal capillary rarefaction, and tubular degeneration ultimately lead to irreversible nephron loss (Sato and Yanagita 2018; Mulay et al. 2012; Lech et al. 2014; Anders 2014). Eventually, sufficiently severe or long-lasting nephron loss overwhelms the physiological compensatory systems (Fattah et al. 2019) and further progresses to overt CKD and ESRD.

To identify a potential CIRP antagonist, we have previously screened 32 overlapping 15-mer oligopeptides covering the entire human sequence of CIRP and identified C23 as a potential CIRP antagonist (Qiang et al. 2013). C23’s affinity for the TLR4/MD2 complex is one order of magnitude higher than that of CIRP and thus, C23 binds to TLR4 and competitively inhibits CIRP binding to the TLR4 receptor. Subsequently, we have previously shown direct anti-inflammatory effects of C23 in acute inflammatory conditions such as sepsis and hemorrhagic shock (Zhang et al. 2017, 2018; McGinn et al. 2018). These anti-inflammatory effects were mediated by blocking the TLR4 signaling pathway. We have also previously shown in the RIR model that C23 treatment at 8 mg/kg BW, the same dose used in the current study, significantly decreased renal inflammation and subsequent injury at 24 h and showed a survival advantage up to 7 days after RIR suggesting C23 acts as a TLR4 antagonist (McGinn et al. 2018). Therefore, the current study extended our knowledge that a single treatment given at the time of reperfusion at the same dose as previously given led to a decrease in renal fibrosis at day 21 after RIR injury. However, we haven’t conducted the experiment administering C23 to RIR-induced CIRP^−/−^ mice to unveil if C23 exhibits protective effect after RIR independently from the inhibition of eCIRP. Additional experiments are warranted for such findings. However, since TGF-β itself can be anti-inflammatory, to directly show C23’s anti-inflammatory effects while inhibiting TGF-β expression additional experiments should be conducted with C23 treatment at different time points in RIR mice and then measure inflammation markers such as cytokine levels and correlate those levels with TGF-β expression. Nevertheless, our current finding suggests that the protective effect of C23 on RIR-induced CKD could be due to the reduction of AKI intensity at the time of reperfusion leading to reduced inflammation, reduced maladaptive repair process and therefore, less CKD.

There are several limitations to our study. One such limitation is that while we demonstrate that C23 has anti-fibrotic effect on CKD, we haven’t addressed that C23 possess a direct anti-fibrotic effect. Since C23 was administered at the time of reperfusion, it is difficult to distinguish between direct effects of CIRP inhibition on RIR-induced renal fibrosis. By administering C23 at later time points in the future will be able to differentiate the direct vs. indirect antifibrotic effect of C23. Second, we have only examined renal injury in one time point, i.e., 21 days after RIR. We have clearly shown the involvement of eCIRP in RIR-induced renal fibrosis but haven’t evaluated the time course in which RIR-induced CKD occur. AKI survivors are at an increased risk of CKD. Currently it is not clear whether eCIRP is responsible for the AKI to CKD transition and whether C23 protects against this transition. The precise kinetics of eCIRP at other time points following RIR and treatment with C23 at those time points are warranted in the future for such determination. In addition, we have only used a single dose of C23 at the time of reperfusion and achieved a significant but moderate decrease in renal injury. Future studies with C23 given at different times post RIR are needed to determine whether delayed intervention could still prevent RIR-induced renal fibrosis. Then utilizing the same dose and time of treatment, the experiment can be extended beyond 21 days to assess the efficacy of C23 in the chronic nature of RIR-induced CKD in the future.

Third, although we have observed significant renal injury and fibrosis on the 21-day post RIR, GFR, the in vivo measure of renal function, wasn’t changed in this model without the inclusion of the left nephrectomy at 72 h post RIR. Since the GFR was conducted using FITC-sinistrin which would have interfered with the analysis of BUN and Creatinine, we haven’t measured BUN and creatinine from these mice. The discrepancy seen in the data between GFR in the bilateral RIR and serum markers of renal function could be due to the transcutaneous method of measuring GFR which could be less sensitive than the conventional methods. Fourth, a major issue of using peptides as therapeutics is the lack of stability in vivo, due to degradation by proteases. Interestingly, our study indicates that a single dose of C23 produced long lasting benefit in this RIR-induced CKD model. The pharmacodynamics and possible other effects of C23 besides inhibiting eCIRP signaling have not been elucidated. Since C23 is derived from human sequence of CIRP and that it is a small peptide, it is highly unlikely that C23 could have measurable adverse effects in mice or humans. However, like any other small peptide, C23 could be degraded easily in circulation. Therefore, the beneficial effect of C23 could be due to rapid intake of the compound into the tissues. Nevertheless, to further develop C23 as a therapeutic in RIR-induced CKD, modifications such as PEGlyation and/or the delivery of the peptide using nanotechnology are needed prior to conducting additional preclinical studies. However, to develop this as a therapeutic in humans, comprehensive pharmacodynamics and safety studies must be conducted in the future. We have only used male mice for the study because our prior studies in RIR were conducted in mice. We acknowledge that the use of male mice is another limitation of this study.

## Conclusions

In summary, we initially demonstrate the critical role of eCIRP in RIR-induced renal fibrosis: the protection of CIRP-deficient mice from renal fibrotic accumulation in this RIR-induced CKD model. Furthermore, we found that the blockade of eCIRP with C23 provides protection against chronic renal injury in this RIR-induced CKD model in mice. We have also demonstrated that intervention with C23 prevented RIR-associated renal fibrotic formation, as evidenced by decreases in the fibrotic factors, i.e., TGF-β1, collagen, fibronectin and α-SMA, and reduction of macrophage infiltration to the kidneys. Finally, we reveal that the administration of C23, an eCIRP competitive antagonist, significantly reduced renal iron accumulation, increased GPX4 expression and reduced lipid peroxidation, thereby attenuating RIR-induced renal ferroptosis. Thus, C23 may serve as a strong candidate for future clinical development aimed at ameliorating RIR-induced renal chronic inflammation, fibrosis, and ferroptosis.

## Data Availability

Data are provided within the manuscript or supplementary information files.

## Abbreviations

CKD: Chronic Kidney Disease
eCIRP: Extracellular Cold Inducible RNA Binding Protein
RIR: Renal Ischemia Reperfusion
TGF-β1: Transforming Growth Factor-Beta 1
EMT: Epithelial-Mesenchymal Transition
GFR: Glomerular Filtration Rate
α-SMA: alpha-smooth muscle actin
GPX4: Glutathione peroxidase 4
CIRP: Cold Inducible RNA Binding Protein Knockout
ESRD: End Stage Renal Disease
ECM: Extracellular Matrix
TLR4/MD2: Toll-Like Receptor/Myeloid Differentiation-2
TREM1: Triggering Receptor Expressed on Myeloid 1
RCD: Regulatory Cell Death
WT: Wild Type
AKI: Acute kidney Injury
ROS: Reactive Oxygen Species
BUN: Blood Urea Nitrogen
NGAL: Neutrophil Gelatinase-Associated Lipocalin
